# Repair of Hypoxanthine in DNA Revealed by DNA Glycosylases and Endonucleases From Hyperthermophilic Archaea

**DOI:** 10.3389/fmicb.2021.736915

**Published:** 2021-08-31

**Authors:** Tan Lin, Likui Zhang, Mai Wu, Donghao Jiang, Zheng Li, Zhihui Yang

**Affiliations:** ^1^College of Environmental Science and Engineering, Marine Science and Technology Institute, Yangzhou University, Yangzhou, China; ^2^Guangling College, Yangzhou University, Yangzhou, China; ^3^College of Plant Protection, Agricultural University of Hebei, Baoding, China

**Keywords:** hyperthermophilic archaea, DNA glycosylase, endonuclease, hypoxanthine, base deamination

## Abstract

Since hyperthermophilic Archaea (HA) thrive in high-temperature environments, which accelerate the rates of deamination of base in DNA, their genomic stability is facing a severe challenge. Hypoxanthine (Hx) is one of the common deaminated bases in DNA. Generally, replication of Hx in DNA before repaired causes AT → GC mutation. Biochemical data have demonstrated that 3-methyladenine DNA glycosylase II (AlkA) and Family V uracil DNA glycosylase (UDG) from HA could excise Hx from DNA, thus triggering a base excision repair (BER) process for Hx repair. Besides, three endonucleases have been reported from HA: Endonuclease V (EndoV), Endonuclease Q (EndoQ), and Endonuclease NucS (EndoNucS), capable of cleaving Hx-containing DNA, thereby providing alternative pathways for Hx repair. Both EndoV and EndoQ could cleave one DNA strand with Hx, thus forming a nick and further initiating an alternative excision repair (AER) process for the follow-up repair. By comparison, EndoNucS cleaves both strands of Hx-containing DNA in a restriction endonuclease manner, thus producing a double-stranded break (DSB). This created DSB might be repaired by homologous recombination (HR) or by a combination activity of DNA polymerase (DNA pol), flap endonuclease 1 (FEN1), and DNA ligase (DNA lig). Herein, we reviewed the most recent advances in repair of Hx in DNA triggered by DNA glycosylases and endonucleases from HA, and proposed future research directions.

## Introduction

DNA is constantly damaged by endogenous and environmental factors. Base deamination is a typical pathway for producing DNA damage. Generally, adenine, cytosine, and guanine are deaminated to hypoxanthine (Hx), uracil (U), and xanthine (X), respectively. Base deamination can occur spontaneously under physiological conditions, and are also accelerated by ionizing radiation, high temperature, aerobic respiration, and nitrosative stress (Chatterjee and Walker, 2017). Since they are mutagenic, deaminated bases in DNA need to be repaired to maintain cellular genomic DNA stability.

Hyperthermophilic Archaea (HA) can grow optimally above 80°C (Stetter, 2013), which are predominantly isolated from high temperature environments, such as deep-sea hydrothermal vents, volcanic craters, and terrestrial hot springs (van der Oost et al., 1998). HA have become a research hotspot because they possess a large number of unknown biological processes and a variety of highly thermostable enzymes that can be potentially applied in biotechnology (Cabrera and Blamey, 2018), and are a simplified model organism for uncovering eukaryotic DNA replication and repair mechanisms (Zatopek et al., 2018). Since high temperature accelerates the rates of base deamination (Lindahl and Nyberg, 1974), HA might have higher levels of U and Hx in their genomic DNA than mesophiles, since they thrive in high temperature environments. Surprisingly, HA display spontaneous mutational frequencies similar to mesophiles (Grogan et al., 2001), thereby suggesting that they possess more efficient repair capability than mesophiles. However, our understanding on DNA damage repair of HA remains incomplete.

Hypoxanthine is a common DNA damage base, which can be formed by two pathways: one is deamination of adenine in DNA, and the other is dHxTP (a product of dATP deamination) incorporation by DNA polymerase (DNA pol). In the first pathway, an Hx/C pairing would be formed after a round of DNA replication, and then a G/C pairing would be produced after the next round of replication, thereby leading to AT→GC mutation (Kuraoka, 2015). In the second pathway, dHxTP can be incorporated opposite the DNA template C since Hx can pair with cytosine, thus forming Hx in DNA. Although the Hx/C pairing in DNA is no-mutagenic (Budke and Kuzminov, 2006), a large amount of Hx accumulation in DNA might trigger the activity of repair-related enzymes, such as EndoV, further causing single-strand breaks (Kuraoka, 2015). Therefore, Hx in DNA is harmful to cells, which needs to be repaired.

Fortunately, cells have evolved several DNA glycosylases, including 3-methyladenine DNA glycosylase II (AlkA), Family V uracil DNA glycosylase (UDG), and Family VI UDG, capable of excising Hx from DNA, thus initiating a typical base excision repair (BER) process to repair Hx in DNA.

Genomic analyses have shown that AlkA and Family V UDG are encoded in HA. Additionally, three endonucleases have been reported from HA: Endonuclease V (EndoV), Endonuclease Q (EndoQ), and Endonuclease NucS (EndoNucS), which are able to cleave Hx-containing DNA, thereby providing alternative pathways for repair of Hx in DNA. Herein, we focused on the most advances of repair of Hx in DNA triggered by two DNA glycosylases (AlkA and Family V UDG) and three endonucleases (EndoV, EndoQ, and EndoNucS) from HA. Furthermore, we proposed a few prospects for future research directions.

## Excision of Hx From DNA by Archaeal AlkA

**Figure 1:**
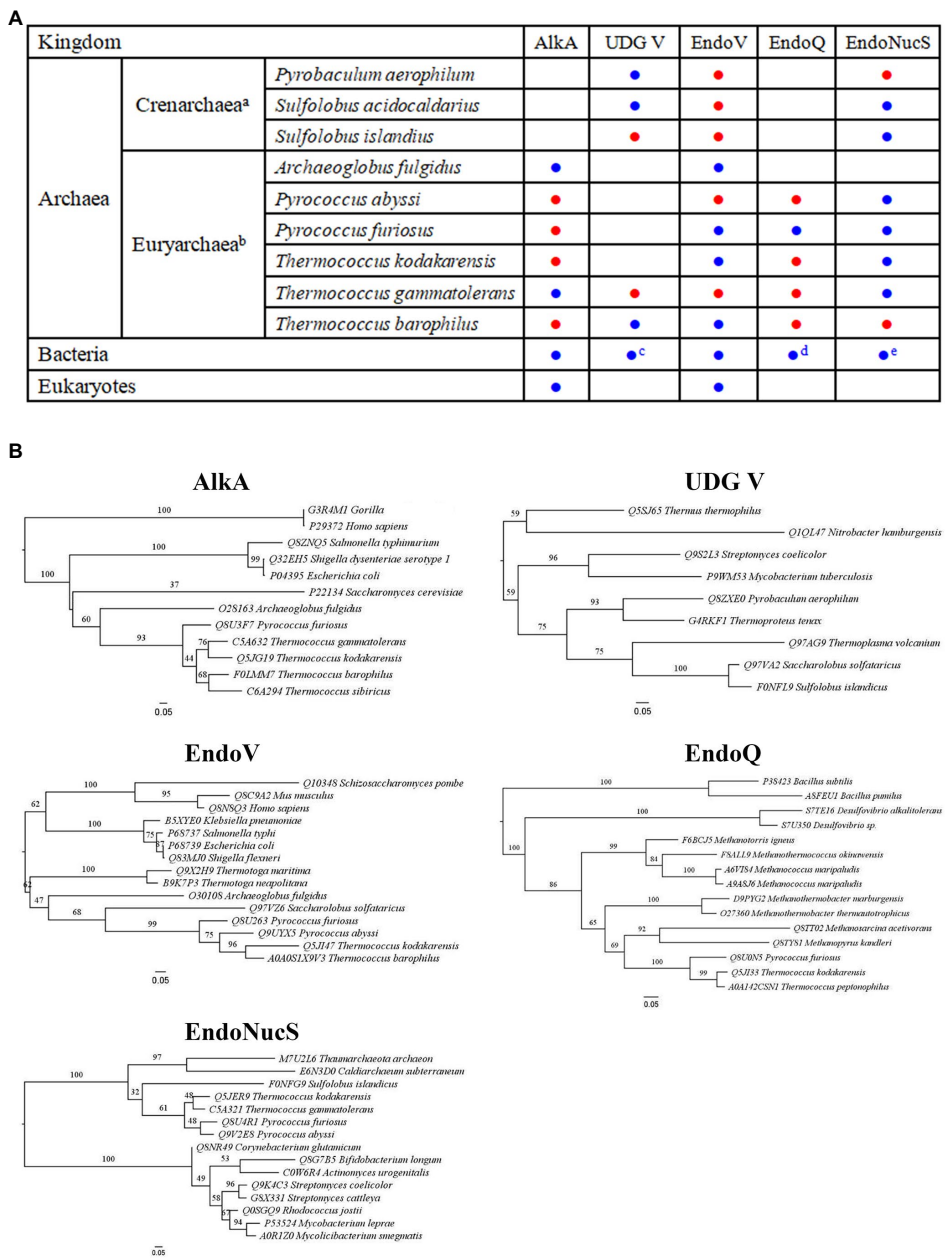
Distribution and phylogenetic analyses of DNA repair proteins involved in Hypoxanthine (Hx) repair. **(A)** Distribution of 3-methyladenine DNA glycosylase II (AlkA), uracil DNA glycosylase (UDG) V, Endonuclease V (EndoV), Endonuclease Q (EndoQ), and Endonuclease NucS (EndoNucS). a and b: only includes archaea which at least one enzyme among five target enzymes has been characterized; c: includes a few hyperthermophilic bacteria; d: includes partial bacteria, such as *Bacillus* and *Desulfovibrio*; and e: includes partial bacteria that lack mismatch repair pathway, such as *Actinobacteria*. The characterized and uncharacterized enzymes are shown with blue and red circle, respectively. **(B)** Phylogenetic analyses of AlkA, UDG V, EndoV, EndoQ, and EndoNucS from hyperthermophilic Archaea. Note that only partial Archaea, bacteria, and eukaryotes are included in these five phylogenetic trees due to the space limit.

**Figure 2:**
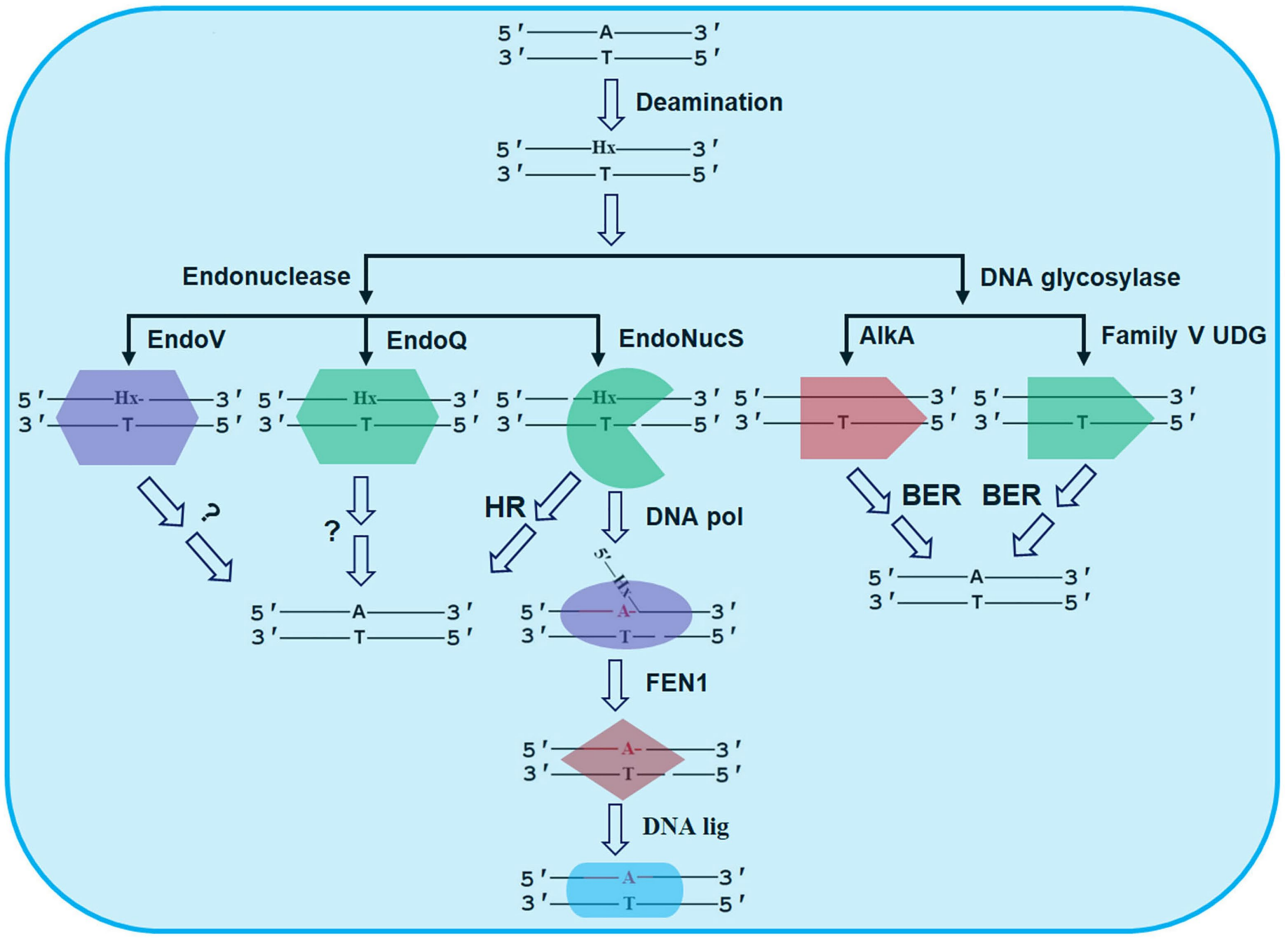
Possible repair pathways of Hx in DNA revealed by DNA glycosylases and endonucleases from HA. The details are seen the text. AlkA, 3-methyladenine DNA glycosylase II; BER, base excision repair; DNA lig, DNA ligase; DNA pol, DNA polymerase; EndoNucS, endonuclease NucS; EndoQ, endonuclease Q; EndoV, endonuclease V; FEN1, flap endonuclease 1; HR, homologous recombination; and UDG, uracil DNA glycosylase.

Base excision repair, which is triggered by DNA glycosylase, is thought to be an important pathway for repair of Hx in DNA (Wallace, 2014). AlkA is a DNA glycosylase that is able to remove methylated bases from DNA. Analysis of genomic sequences has shown that AlkA is ubiquitous in most of bacteria and eukaryotes, and a few Euryarchaea rather than Crenarchaea (Figure 1A). In addition to removal of methylated bases from DNA, AlkA can also possess the activity toward excising Hx from DNA (Figure 2; Evensen and Seeberg, 1982). A number of structural and biochemical studies have revealed damaged base recognition and removal mechanisms of *Escherichia coli* AlkA (Nakabeppu et al., 1984; Bjelland and Seeberg, 1996; Labahn et al., 1996; Yamagata et al., 1996; O’Brien and Ellenberger, 2004), thus enabling the enzyme to be a model alkylated DNA glycosylase. Currently, only two archaeal AlkA homologs have been reported from the hyperthermophilic euryarchaeon *Archaeoglobus fulgidus* and *Thermococcus gammatolerans* (Figure 1; Birkeland et al., 2002; Mansfield et al., 2003; Leiros et al., 2007; Jiang et al., 2021). Thus, our understanding on biochemical function and catalytic mechanism of archaeal AlkA remains incomplete.

Biochemical data have demonstrated that *A. fulgidus* AlkA and *T. gammatolerans* AlkA are able to remove Hx from dsDNA (Mansfield et al., 2003; Jiang et al., 2021). Although, the two protein hosts are hyperthermophiles, *T. gammatolerans* AlkA displays biochemical characteristics distinct from *A. fulgidus* AlkA. Firstly, *T. gammatolerans* AlkA is able to excise Hx from dsDNA rather than from ssDNA (Jiang et al., 2021), suggesting that the enzyme might be critical for repair of Hx in genomic DNA. By comparison, *A. fulgidus* AlkA can remove Hx from ssDNA (Mansfield et al., 2003). Additionally, *T. gammatolerans* AlkA is a bi-functional DNA glycosylase, harboring the activity for not only excising Hx from dsDNA but further cleaving the generated apurinic/apyrimidinic (AP) site (Jiang et al., 2021), while *A. fulgidus* AlkA is monofunctional DNA glycosylase, only removing Hx from DNA (Mansfield et al., 2003). Overall, effective excision of Hx from DNA by the two archaeal AlkA proteins might provide an alternative pathway for repair of Hx in DNA.

Analysis of amino acid sequences of AlkA homologs suggests that these AlkA proteins harbor a conserved helix-hairpin-helix (HhH)-GPD motif that is composed of an HhH motif for binding DNA and a glycine/proline-rich loop followed by an invariable Asp (GPD; Kuznetsov and Fedorova, 2020). Mutational analyses have demonstrated that the conserved Asp residue in the HhH-GPD motif serves as a catalytic residue in *T. gammatolerans* AlkA and *A. fulgidus* AlkA (Mansfield et al., 2003; Jiang et al., 2021), which is also observed in *Helicobacter pylori* AlkA (Eichman et al., 2003). Additionally, residue W204 in *T. gammatolerans* AlkA is another essential residue for catalysis since the replacement of residue W204 with alanine abolishes the enzyme activity (Jiang et al., 2021). Note that residue W204 in *T. gammatolerans* AlkA corresponds to residue W218 in *E. coli* AlkA. The mutant *E. coli* cells harboring the AlkA W218A mutant display the sensitivity to methylmethane sulfonate (MMS), whereas the wild-type cells are resistant to MMS, suggesting that residue W218 is part of the active site residues of the enzyme (Yamagata et al., 1996). The crystal structure of *E. coli* AlkA demonstrates that residue W218 is positioned behind the ribose of the flipped out the damaged base for the attack on the back of the glycosylic bond (Yamagata et al., 1996; Hollis et al., 2004).

Genetic studies suggest that *Pseudomonas putida* AlkA plays an essential role in repairing induced lesions by MMS or N-methyl-N'-nitro-N-nitrosoguanidine *in vivo* (Mielecki et al., 2013). Additionally, the exposure to alkylating agents leads to about 100-fold increase in the *E. coli alkA* gene transcription (Samson and Cairns, 1977; Evensen and Seeberg, 1982; Nakabeppu et al., 1984), suggesting that induced AlkA is involved in repair of alkylation lesion *in vivo*. Similar effect is observed in *Saccharomyces cerevisiae* AlkA (Chen et al., 1990). However, no genetic analysis on archaeal AlkA has been currently reported.

## Excision of Hx From DNA by Archaeal Family V UDG

Uracil DNA glycosylase is thought to be responsible for repair of uracil in DNA. Based on amino acid sequence similarity, UDG is classified into six families, and each family possesses specific characteristics (Schormann et al., 2014). Hx DNA glycosylase, which belongs to Family VI UDG, was first reported from the archaeon *Methanosarcina barkeri*, capable of excising Hx from DNA (Lee et al., 2011). However, no Hx DNA glycosylase homolog has been found in HA to date.

Family V UDG is present in hyperthermophilic bacteria, such as *Thermus* and *Mycobacterium*, and hyperthermophilic Crenarchaea and Euryarchaea (Figure 1B; Kosaka et al., 2007; Xia et al., 2014), capable of removing Hx from DNA in addition to uracil excision (Figure 2). At least three different UDGs are encoded in the hyperthermophilic crenarchaeon *Pyrobaculum aerophilum*: Pa-MIG (mismatch DNA glycosylase), Pa-UDGa, and Pa-UDGb. Pa-UDGb was the first reported as an archaeal Family V UDG member (Sartori et al., 2002), displaying the activity toward removal of Hx from DNA in addition to uracil, hydroxymethyluracil, and fluorouracil opposite from guanine as well. Thus, removal of Hx from DNA by *P. aerophilum* UDGb might potentially function in avoiding A to G transition mutations in the archaeal cells, which needs to be confirmed *in vivo*.

The Family V UDG from *Thermus thermophilus* HB8 is capable of removing uracil, hypoxanthine and xanthine from DNA (Xia et al., 2014). Genetic studies suggest that *T. thermophilus* UDG plays a crucial role removing uracil from DNA *in vivo* (Sakai et al., 2008). Additionally, the *udgB* knockout strain from *Mycobacterium smegmatis* shows approximately 2-fold higher mutation rates than the wild-type strain, suggesting that *M. smegmatis* UDGb is involved in repair of deaminated bases. Further genetic studies suggest a synergistic effect of *M. smegmatis* UDGb and UNG (uracil N-glycosylase, Family I UDG) in DNA repair (Malshetty et al., 2010). Currently, no archaeal family V UDG function has been described *in vivo*.

## Cleavage of Hx-Containing DNA by Archaeal EndoV

In addition to BER, alternative excision repair (AER) is also thought to be another important pathway for repair of Hx in DNA. AER is generally initiated by an endonuclease that can nick DNA (Yasui, 2013). However, the complete AER pathway has not been clarified to date. EndoV is the first reported endonuclease that triggers the AER process for repair of Hx in DNA (Cao, 2013).

Endonuclease V is a DNA repair enzyme encoded by the *nfi* gene, which was first reported in *E. coli* (Eco-EndoV), capable of cleaving the second phosphodiester bond downstream of the deaminated damaged base (Figure 2; Gates and Linn, 1977; Demple and Linn, 1982). EndoV is ubiquitously present in three domains: bacteria, Archaea, and eukaryotes (Figure 1B). Alignment of amino acid sequences of EndoV homologs from bacteria, Archaea, and eukaryotes has shown that EndoV has seven conserved motifs (Wang et al., 2018), suggesting similar catalytic mechanism.

Since the hyperthermophilic euryarchaeon *A. fulgidus* EndoV (Afu-EndoV) was first reported (Liu et al., 2000), two other archaeal EndoV homologs have been characterized biochemically, including the hyperthermophilic euryarchaeon *Pyrococcus furiosus* EndoV (Pfu-EndoV; Kiyonari et al., 2014), and *Thermococcus barophilus* Ch5 EndoV (Tba-EndoV; Wang et al., 2018). No relevant structure of archaeal EndoV has been solved to date. Afu-EndoV displays 39% amino acid sequence identity and 55% similarity with Eco-EndoV (Liu et al., 2000). Whereas Eco-EndoV cleaves DNA substrates with other damaged bases in addition to Hx (Demple and Linn, 1982), Afu-EndoV only cleaves DNA containing Hx (Liu et al., 2000). Overall, cleaving Hx-containing DNA is the most primitive activity of EndoV homologs from bacteria and Archaea.

Biochemical data demonstrate that three archaeal EndoV homologs are able to cleave Hx-containing DNA at high temperature, and their optimal reaction temperature are 70~90°C. However, these three archaeal EndoV homologs display distinct biochemical characteristics. Firstly, Pfu-EndoV can not only cleave Hx-containing DNA, but also cleave Hx-containing RNA (Kiyonari et al., 2014), suggesting that this enzyme may be involved in RNA metabolism. By comparison, Afu-EndoV and Tba-EndoV act on only Hx-containing DNA (Liu et al., 2000; Wang et al., 2018). Secondly, Tba-EndoV can bind normal DNA and Hx-containing DNA, and display higher binding Hx-containing DNA (Wang et al., 2018), while Pfu-EndoV can only bind Hx-containing DNA (Kiyonari et al., 2014). Furthermore, Pfu-EndoV is inactive to cleave DNA at pH 6.0 (Kiyonari et al., 2014), while Tba-EndoV still possesses strong cleavage activity at pH 6.0 (Wang et al., 2018). Additionally, Tba-EndoV is more resistant to NaCl than Pfu-EndoV (Kiyonari et al., 2014; Wang et al., 2018).

Genetic studies suggest that Eco-EndoV plays a significant role in repair of Hx and abasic site in DNA (Guo and Weiss, 1998). Compared with the wild-type, the *nfi* mutant of *E. coli* displays about 8-fold lower Hx repair levels *in vivo*, and the exonuclease activity of *E. coli* DNA polymerase I is required for the Eco-EndoV repair pathway (Su et al., 2018). Additionally, the analysis of effect of EndoV deletion in *Bacillus subtilis* on spontaneous mutation rates suggests the presence of this pathway *in vivo*, which counteracts the genotoxic effects of base deamination (Patlán et al., 2019). To data, no genetic study on archaeal EndoV function has been currently reported.

## Cleavage of Hx-Containing DNA by EndoQ From HA

In addition to EndoV, a second endonuclease was recently identified in *P. furiosus* as Pfu-EndoQ, capable of cleaving damaged DNA, including U, Hx, and AP site (Shiraishi et al., 2015). Pfu-EndoQ is able to recognize and cleave the first phosphodiester bond on the 5'-side of damaged bases in DNA to form a nick (Figure 2; Shiraishi et al., 2015). Recently, molecular mechanism of recognition of damaged bases in DNA by Pfu-EndoQ was revealed, demonstrating that the damaged bases are flipped out from the DNA duplex by the enzyme, thus leading to the base pair instability of the damaged bases and further enhancing the cleavage activity of the enzyme (Shiraishi and Iwai, 2020).

Genomic analyses show that EndoQ are only found in Euryarchaea and a few bacteria including *Bacillus* and *Desulfovibrio* (Figure 1B), whereas EndoV is widespread in all organisms. The limited distribution of EndoQ suggests that the enzyme is involved in the specific repair pathway of damaged bases. Although EndoQ appears to be conserved in a limited number of species, the substrate specificities of the EndoQ homologs seem to be highly conserved. The archaeal EndoQ homologs from *Thermococcus kodakarensis* and the methanogen *Methanosarcina acetivorans* and the bacterial EndoQ homolog from *Bacillus pumilus* have been shown to create a nick on DNA strands containing uracil, Hx, and AP site (Shiraishi et al., 2015, 2017, 2018).

Further biochemical data have shown that Pfu-EndoQ interacts with the sliding clamp proliferating cell nuclear antigen (PCNA) protein (Shiraishi et al., 2016), a central protein, which coordinates DNA replication and DNA repair, and other biological processes (Moldovan et al., 2007), which may help to recruit the enzymes or proteins to the replication fork. The nick created by the enzyme is repaired by a combination activity of DNA helicase and endonuclease flap endonuclease 1 (FEN1), DNA pol, and DNA ligase (DNA lig). Additionally, the Pfu-EndoQ activity is stimulated by PCNA, and the physical interaction has been confirmed between the two proteins through a PIP-motif of EndoQ and the toroidal structure of PCNA, thus providing a clue to elucidate a unique DNA repair system in Archaea (Shiraishi et al., 2016).

EndoV cleaves the second phosphodiester bond downstream of Hx in DNA, while EndoQ cleaves the first phosphodiester bond on the 5'-side of Hx in DNA (Demple and Linn, 1982; Shiraishi et al., 2015), thereby suggesting that Hx in DNA might be repaired by EndoV and EndoQ together. However, no direct interaction is confirmed between EndoQ and EndoV in *P. furiosus* no matter whether Hx-containing DNA is present or not (Ishino et al., 2015). Compared with Pfu-EndoV, Pfu-EndoQ has a higher affinity for DNA containing Hx. In addition, the expression levels of Pfu-EndoQ in *P. furiosus* cells are higher than those of Pfu-EndoV, suggesting that Pfu-EndoQ might participate in repair of Hx in DNA *in vivo*.

Recently, the crystal structures of *P. furiosus* EndoQ bound to DNA substrates containing U, Hx, or an AP lesion have been solved (Shi et al., 2021), demonstrating the mechanisms of recognition and cleavage of deaminated bases in DNA by this endonuclease. The structures of the *P. furiosus* EndoQ-DNA complexes show that a deep active-site pocket in the enzyme would be shaped by a concerted swing motion of its zinc-binding and C-terminal helical domains (Shi et al., 2021), which allows the extruded deaminated bases to be accommodated. Furthermore, U and Hx bases associate with amino acid residues in this pocket, which is coordinated with an essential magnesium ion (Shi et al., 2021). Thus, the EndoQ-DNA complex structures provide mechanistic insights into damaged DNA recognition and cleavage by this endonuclease, which is helpful for understanding how EndoQ recognizes and cleaves these structurally diverse damaged DNA substrates rather than undamaged DNA substrate.

## Cleavage of Hx-Containing dsDNA by EndoNucS From HA

Endonuclease NucS was identified from HA as the third endonuclease, capable of cleaving Hx-containing DNA. EndoNucS was the first reported from the hyperthermophilic euryarchaeon *Pyrococcus abyssi* (Ren et al., 2009), displaying the activity on branched and splayed DNA. Later, the EndoNucS homologs from the hyperthermophilic euryarchaeon *T. kodakarensis* and *T. gammatolerans*, and the hyperthermophilic crenarchaeon *Sulfolobus islandius* REY15A have been reported, possessing the activity on mismatched and Hx-containing DNA (Ishino et al., 2016; Ahmad et al., 2020; Zhang et al., 2020a). Intriguingly, these three EndoNucS homologs from HA harbor distinct cleavage sites, as reviewed in our recent publication (Zhang et al., 2020b).

Biochemical data suggest that these three reported EndoNucS homologs from HA cleave both strands of Hx-containing dsDNA in a restriction endonuclease manner, thereby forming a double-stranded break (DSB), which is sharply distinct from EndoQ and EndoV as discussed above. Since they might pose a severe damage for archaeal cells, the DSBs potentially generated by archaeal EndoNucS need to be repaired by homologous recombination (HR) or a combinational activity of DNA pol, FEN1, and DNA lig (Figure 2). These hypotheses need to be verified by genetic analysis *in vivo*. Overall, DNA cleavage by archaeal EndoNucS provides a possible alternative pathway for repair of Hx in DNA.

Endonuclease NucS is distributed in Euryarchaea, Crenarchaea, and a few bacteria, especially the bacteria that lack a typical mismatch repair pathway, such as *Actinobacteria* (Figure 1B). The analysis of amino acid sequences of EndoNucS homologs suggests that this endonuclease possesses several conserved motifs that are composed of mostly negatively and positively charged amino acid residues (Zhang et al., 2020a). Structural and mutational studies have provided insight into catalytic mechanism of archaeal EndoNucS. The complex structure of *T. kodakarensis* EndoNucS dimer with mismatched dsDNA was solved (Nakae et al., 2016), demonstrating that the mismatched bases are flipped out into binding sites, which resembles the overall architecture of most restriction endonucleases.

Mutational data have demonstrated that residues Y41, N76, and W77 are essential for DNA binding, and residues D165, E179, and K181 are critical for catalysis (Ishino et al., 2016). Additionally, we revealed that the replacement of residue D163 in *T. gammatolerans* EndoNucS, which is analogous to residue D165 in *T. kodakarensis* EndoNucS, with alanine leads to the partial loss of the enzyme activity on U- and Hx-containing dsDNA, suggesting that residue D163 is essential for catalysis (Zhang et al., 2020a).

Genetic data have demonstrated that the *nucS* deletion strain from the actinobacterium *Corynebacterium glutamicum* leads to a drastic increase of spontaneous transition mutations, thereby suggesting that *C. glutamicum* EndoNucS protein plays an essential role in repair mismatch (Ishino et al., 2018). Similar effects are observed in *M. smegmatis*, *Streptomyces coelicolor*, and *S. islandicus* REY15A (Castaneda-Garcia et al., 2017; Takemoto et al., 2018; Ahmad et al., 2020). By comparison, the *nucS* deletion strain from *S. acidocaldarius* does not cause the increased mutation rates (Suzuki and Kurosawa, 2019). Further genetic data suggest that this endonuclease associates with XPF endonuclease *via* the HR-mediated stalled-fork to remove helix-distorting DNA lesions, such as intrastrand crosslinks (Suzuki and Kurosawa, 2019). Thus, more work needs to be done to investigate the archaeal EndoNucS function *in vivo*.

## Conclusion and Future Directions

Adenine deamination produces Hx, which is one of common deamination types. Since it is mutagenic, Hx in DNA needs to be repaired. HA encodes AlkA and Family V UDG, triggering the BER process for repairing Hx in DNA. Besides, some HA encode three endonucleases: EndoV, EndoQ, and EndoNucS, which has been confirmed that they are capable of cleaving Hx-containing DNA. Interestingly, these archaeal endonucleases cleave Hx-containing DNA in distinct manners. Overall, a combination of these potential repair pathways initiated with DNA glycosylases and endonucleases might enable HA to counteract the potentially increased mutations caused by high temperature, thus maintaining spontaneous mutation frequencies similar to other mesophiles.

AlkA and Family V UDG from HA can excise Hx from DNA *in vitro*. However, their physiological function *in vivo* still remains unclear. Whether AlkA or Family V UDG is a major DNA glycosylase for repair of Hx in DNA in archaeal cell is unknown. A combination of structural, biochemical, and genetic analyses for these two DNA glycosylases would provide new insights into Hx repair in HA.

Archaeal EndoV and EndoQ cleave Hx-containing DNA, thus forming a nick. Generally, the 3’-OH of the nick generated by archaeal EndoV and EndoQ can be utilized by other nucleic acid enzymes to complete subsequent repairs. Recently, EndoV, ExoA, and PolA are jointly involved in repair of DNA containing deaminated bases in *B. subtilis* (Patlán et al., 2019). However, how the nicks created by archaeal EndoV and EndoQ are repaired remains elusive. It would be interesting to investigate molecular mechanism of repair of Hx in DNA triggered by EndoV and EndoQ *in vivo*.

As discussed above, the EndoNucS homologs from the hyperthermophilic *T. kodakarensis*, *S. islandius*, and *T. gammatolerans* are able to cleave Hx-containing dsDNA in a restriction endonuclease manner *in vitro*, leading to the formation of DSB. The evidence for the generated DSBs by archaeal EndoNucS needs to be provided to confirm whether the DSB is in fact generated *in vivo* and the nuclease activity is directed in a strand-specific manner similar to MutL proteins in Bacteria.

## Author Contributions

All authors listed have made a substantial, direct and intellectual contribution to the work, and approved it for publication.

## Conflict of Interest

The authors declare that the research was conducted in the absence of any commercial or financial relationships that could be construed as a potential conflict of interest.

## Publisher’s Note

All claims expressed in this article are solely those of the authors and do not necessarily represent those of their affiliated organizations, or those of the publisher, the editors and the reviewers. Any product that may be evaluated in this article, or claim that may be made by its manufacturer, is not guaranteed or endorsed by the publisher.
